# Novel cholesterol‐dependent regulation of cardiac K_ATP_ subunit expression revealed using histone deacetylase inhibitors

**DOI:** 10.14814/phy2.14675

**Published:** 2020-12-23

**Authors:** Robert Geiger, Naheed Fatima, James F. Schooley, Jeremy T. Smyth, Mark C. Haigney, Thomas P. Flagg

**Affiliations:** ^1^ Department of Anatomy, Physiology, and Genetics Uniformed Services University for the Health Sciences Bethesda MD USA; ^2^ Department of Medicine Uniformed Services University for the Health Sciences Bethesda MD USA

**Keywords:** Abcc9, SREBP, SUR2, trichostatin A

## Abstract

We recently discovered that the histone deacetylase inhibitor, trichostatin A (TSA), increases expression of the sulfonylurea receptor 2 (SUR2; *Abcc9*) subunit of the ATP‐sensitive K^+^ (K_ATP_) channel in HL‐1 cardiomyocytes. Interestingly, the increase in SUR2 was abolished with exogenous cholesterol, suggesting that cholesterol may regulate channel expression. In the present study, we tested the hypothesis that TSA increases SUR2 by depleting cholesterol and activating the sterol response element binding protein (SREBP) family of transcription factors. Treatment of HL‐1 cardiomyocytes with TSA (30 ng/ml) caused a time‐dependent increase in SUR2 mRNA expression that correlates with the time course of cholesterol depletion assessed by filipin staining. Consistent with the cholesterol‐dependent regulation of SREBP increasing SUR2 mRNA expression, we observe a significant increase in SREBP cleavage and translocation to the nucleus following TSA treatment that is inhibited by exogenous cholesterol. Further supporting the role of SREBP in mediating the effect of TSA on K_ATP_ subunit expression, SREBP1 significantly increased luciferase reporter gene expression driven by the upstream SUR2 promoter. Lastly, HL‐1 cardiomyocytes treated with the SREBP inhibitor PF429242 significantly suppresses the effect of TSA on SUR2 gene expression. These results demonstrate that SREBP is an important regulator of K_ATP_ channel expression and suggest a novel method by which hypercholesterolemia may exert negative effects on the cardiovascular system, namely, by suppressing expression of the K_ATP_ channel.

## Graphical abstract

The ATP‐sensitive potassium (KATP) channel can protect the heart during metabolic challenges. Our study shows that SREBP is a regulator of cardiac KATP channel expression and suggests a novel method by which hypercholesterolemia may exert negative effects on the cardiovascular system, namely, by suppressing expression of the KATP channel.

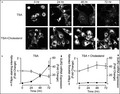

**New and Noteworthy**

ATP‐sensitive potassium (K_ATP_) channels play a variety of roles in the cardiovascular system. They protect cardiomyocytes during metabolic stress and help regulate vascular smooth muscle tone. In this study, we show that transcription of SUR2, a key K_ATP_ subunit in the cardiovascular system, is regulated by the cholesterol‐sensitive sterol response element binding protein (SREBP) signaling pathway. This suggests a novel way that hypercholesterolemia may affect cardiovascular health and function.

## 1 INTRODUCTION

It is estimated that about 30 million adults live with high serum cholesterol levels (≥240 mg/dl) (Benjamin et al., 2019). High cholesterol has been associated with poor cardiovascular health. Elevated serum cholesterol is correlated with atherosclerosis and significant morbidity and mortality (Parish et al., 2012), however, vascular pathology alone does not fully explain the benefits of cholesterol‐lowering therapies in reducing the risk of developing fatal arrhythmias that originate in the heart (Buber et al., 2012). Studies have shown that a high cholesterol diet is associated with an increase in intercellular cholesterol in cardiac myocytes with effects on intracellular Ca^2+^ cycling (Huang et al., 2004; Jacobson et al., 1985), but it is not entirely clear whether elevated cholesterol affects other aspects of cardiac physiology.

ATP‐sensitive potassium (K_ATP_) channels are known to play an important role in the heart during metabolic stress (Flagg et al., 2010; Foster & Coetzee, 2016). K_ATP_ channels are activated when intracellular ATP concentration falls or MgADP rises (Lederer & Nichols, 1989; Nichols & Lederer, 1990; Noma, 1983). During myocardial ischemia, K_ATP_ activation serves to shorten the cardiac action potential, reduce Ca^2+^ entry into the cell, and cause a characteristic ST‐segment elevation in the ECG (Baczkó et al., 2005; Kubota et al., 1993; Li et al., 2000; Nichols & Lederer, 1990). Inhibition of K_ATP_ with sulfonylureas or by genetic deletion of subunits causes increased myocardial damage following ischemia and reperfusion (Cole et al., 1991; Suzuki et al., 2002). Conversely, increasing K_ATP_ activity using pinacidil or by overexpressing ion channel subunits is protective against ischemia reperfusion injury (Cole et al., 1991; Du et al., 2006). Sarcolemmal K_ATP_ channels are also essential for ischemic preconditioning where brief bouts of ischemia serve to protect the heart during a subsequent long‐term ischemic event (Gross & Auchampach, 1992; Wojtovich et al., 2013). Given the importance of K_ATP_ in response to cardiac stress, adjusting its expression is expected to have consequences on cardiovascular health.

In the mouse heart, the sarcolemmal K_ATP_ channel is a hetero‐octomeric protein complex composed of four inward rectifier (Kir6.2; *Kcnj11*) pore‐forming subunits combined with four sulfonylurea receptor subunits (SURx; *Abccx)* (Chutkow et al., 2001; Li et al., 2000; Suzuki et al., 2001). In mouse atrial cardiomyocytes, SUR1 (*Abcc8*) is the sulfonylurea receptor present, while in ventricular myocytes, SUR2A (*Abcc9*) is the major accessory subunit (Chutkow et al., 2001; Flagg et al., 2008). We recently showed that the canonical histone deacetylase inhibitor (HDACi), trichostatin A (TSA), causes a significant increase in the expression of SUR2 in HL‐1 cardiomyocytes (Fatima et al., 2015).

TSA is typically thought to increase gene expression by inhibiting histone deactylases leading to an increase in histone acetylation that promotes unwinding of chromatin to increase transcription factor accessibility. In our previous study, we found a TSA‐dependent increase in histone H3 acetylation (Lys9) at the SUR2 promoter consistent with this proposed model of epigenetic regulation (Fatima et al., 2015). Surprisingly, we also observed that cellular cholesterol was reduced in TSA‐treated HL‐1 cells and that cholesterol inhibited the effect of TSA on K_ATP_ subunit expression without affecting histone acetylation (Fatima et al., 2015). TSA has been shown to reduce cholesterol accumulation in mutant Neimann Pick Type C1 (NPC) fibroblasts and SH‐SY5Y cells (Nunes et al., 2013; Pipalia et al., 2011). Taken together, these previous results suggest that in addition to increasing histone acetylation, TSA may deplete HL‐1 cells of cholesterol thereby stimulating a cholesterol‐sensitive signaling pathway that regulates K_ATP_ channel expression.

One well‐known cholesterol‐sensitive transcription factor is the sterol response element (SRE) binding protein or SREBP (Horton et al., 2002). When cholesterol levels in the cell are sufficient, SREBP interacts with a protein complex containing sterol cleavage activating protein (SCAP) and Insig, sequestering SREBP in the endoplasmic reticulum (ER). When cholesterol levels in the cell fall, reduced cholesterol in the ER causes the SCAP/SREBP complex to dissociate from Insig and translocate to the Golgi, where SREBP can be cleaved by site‐1 protease (S1P). Cleavage of SREBP allows the transcription factor to translocate to the nucleus, bind SREs, and activate transcription of target genes (Wang et al., 1994). Therefore, a decrease in intracellular cholesterol is predicted to activate SREBP signaling, while an increase in intracellular cholesterol inhibits SREBP‐dependent gene transcription.

There are two different genes encoding SREBP isoforms—SREBP1 and SREBP2 (Brown & Goldstein, 1999). SREBP1 has two splice variants (SREBP‐1a and SREBP‐1c) that are typically associated with regulating the genes required for fatty acid and cholesterol synthesis, while SREBP2 typically regulates expression of enzymes needed for cholesterol synthesis and transport. However, it is increasingly recognized that SREBP proteins can regulate the expression of genes other than those involved in cholesterol or lipid homeostasis. For example, a recent study demonstrates a role for SREBP in regulating the parasympathetic response of chick cardiomyocytes through regulation of G‐protein gated inward rectifying K^+^ channels expression (Park et al., 2008). Interestingly, *in silico* analysis of the SUR2 promoter suggests that it contains a SRE (Philip‐Couderc et al., 2008), leading to the hypothesis that SREBP may be the cholesterol‐sensitive mediator linking TSA treatment, cholesterol depletion, and K_ATP_ subunit expression.

In this study, we tested the hypothesis that TSA increases SUR2 expression through a mechanism that requires cholesterol depletion and involves activation of SREBP signaling. The results show that increased SUR2 expression correlates with decreased levels of cellular cholesterol, cleavage, and translocation of SREBP1. Moreover, we show that inhibition of SREBP signaling suppresses the effects of TSA on gene expression. Taken together, these results reveal a novel cholesterol‐dependent mechanism that regulates K_ATP_ subunit expression and may contribute to poor cardiovascular health associated with hypercholesterolemia.

## 2 MATERIALS AND METHODS

### 2.1 Cell culture

All cells were maintained in a humidified incubator with 5% CO_2_ at 37°C. HL‐1 cardiomyocytes were cultured on tissue culture vessels coated with gelatin (0.02%w/v) and fibronectin (0.5%v/v; Sigma) and were maintained in supplemented Claycomb media as previously described (Elcarpio et al., 1998; Fatima et al., 2012, 2015). COSm6 cells were maintained in Dulbecco's Modified Eagle's Medium (DMEM; Gibco) supplemented with 10% Fetal Bovine Serum and (100 µg/ml) Penicillin/Streptomycin. Cells were passaged using 0.05% trypsin/0.02% EDTA. In some experiments, HL‐1 cells were treated with Claycomb media supplemented with Trichostatin A (TSA), cholesterol (250× cholesterol lipid concentrate, Gibco cat no. 12531‐018), or PF429242 (Sigma cat no. SML0667) at concentrations and durations indicated in the text.

### 2.2 Imaging experiments

In all imaging experiments, HL‐1 cardiomyocytes on glass coverslips were fixed with 4% paraformaldehyde (FD NeuroTechnologies, cat no. PF101), quenched with 0.15% glycine, and labeled for either cholesterol quantitation or SREBP‐1 immunolocalization. Cells examined for cholesterol quantitation were stained with filipin (0.05 mg/ml; Cayman chemical, cat no. 70440), PicoGreen (Invitrogen, cat no. P11496), and FBS 10% in PBS for 2 hr. Cells examined for SREBP‐1 localization were blocked and permeabilized with 10% FBS, 0.1% Trition X‐100 in PBS at RT for 1 hr. Following blocking, cells were incubated with antibodies specific to SREBP‐1 (1:50; Abcam, cat no. ab28481) in 0.1% triton ×100 at 4 degrees overnight. To visualize SREBP‐1 antibody localization, secondary Alexa Fluor 488 conjugated anti‐rabbit IgG 2° antibody (1:500 Invitrogen) with 0.1% triton ×100 and DAPI in PBS at RT for 1 hr. Labeled coverslips were mounted with VECTASHIELD (Vector, cat no. H‐1000) on glass microscope slides and sealed with nail polish. To visualize filipin staining, cells were exposed to UV light (ex: 370–420 nM) and fluorescence (em: 410–460 nM) was collected on a Nikon Eclipse Ti2 microscope using a 40× oil objective. To visualize SREBP‐1 localization, cells were excited at 495 nM and fluorescence was collected on a Nikon A1 resonant scanning confocal system. Image quantitation was performed on reconstructed Z‐projections (0.5 µM between each slice for the z stack) encompassing the entire volume of the cell. Images were quantitated using Fiji and the mean fluorescence intensity was normalized to time 0 hr and the nuclear fluorescence was normalized to control (Schindelin et al., 2012).

### 2.3 Protein isolation and Western blot analysis

To isolate proteins, cells were lysed with RIPA buffer (Sigma, cat no. R0278), supplemented with PMSF and protease inhibitors (Roche, cat no. 11836153001). Protein concentration was estimated using the BCA assay (Thermo Scientific). Protein was separated using polyacrylamide gel electrophoresis (4–15%) and transferred to a PVDF membrane (Bio‐Rad Inc.). The membrane was blocked in 5% milk and incubated with anti‐SREBP antibody (1:250; Santa Cruz, cat no. sc‐365513) overnight at 4 degrees followed by anti‐mouse IgG secondary antibody (cat no. A11008) in TBS‐T for 1 hr. The signal was observed using SuperSignal West Dura Extended Duration substrate (Thermo Scientific) and images captured on the Bio‐Rad ChemiDoc imager and analyzed using Fiji (Schindelin et al., 2012).

### 2.4 Quantitative RT‐PCR

mRNA expression was assessed using qPCR as described previously (Fatima et al., 2015). Briefly, RNA was extracted from HL‐1 cells using Quick‐RNA MiniPrep (Zymo Research) and treated with DNase to remove residual genomic DNA. RNA concentration and quality were determined using a NanoDrop 2000 Spectrophotometer (Nanodrop Technologies Inc.). cDNA was synthesized from 1ug of RNA using the High‐Capacity cDNA Reverse Transcription kit (Applied Biosystems Inc.). qPCR was performed on a CFX384 Real‐Time System C1000 Thermal Cycler (Bio‐Rad Inc.) using Taqman probe and primer pairs obtained from Applied Biosystems, Inc. Reactions were performed in triplicate. Relative fold changes (normalized to *HPRT)* were reported as 2^–(ΔΔCt)^, where ΔΔCt = ΔCt_(drug)_ − ΔCt_(notreatment)_.

### 2.5 Luciferase assay

Transcriptional activation of the SUR2 upstream regulatory sequence by SREBP signaling was assessed using luciferase assays. Briefly, the 470 bp sequence immediately upstream of the SUR2 transcription start site was amplified by PCR and cloned into the pGluc Mini‐TK *Gaussia* luciferase reporter vector (New England Biolabs; cat no. N8086). cDNA encoding SREBP‐1a (pcDNA3.1‐2xFLAG‐SREBP‐1a) was a gift from Timothy Osbourne (Addgene plasmid #26801; http://n2t.net/addgene:26801; RRID:Addgene_26801) (Toth et al., 2004). A predicted SRE, homologous to that found in SREBP target genes like PCSK9 was identified in the SUR2 promoter using Genomatix software (Cartharius et al., 2005). We also deleted three nucleotides (TCA) within the site using the Q5 site‐directed mutagenesis kit (New England BioLabs cat# E0554S) resulting in the construct pGluc SUR2_SRE_Mut‐Luciferase. COSm6 cells were plated in a 24‐well plate at a concentration of 50,000 cells per well. Following overnight incubation, each well was transfected with 500 ng pcDNA3.1‐2xFLAG‐SREBP‐1a and 500 ng pGluc SUR2‐Luciferase or pGluc SUR2_SRE_Mut‐Luciferase using Lipofectamine 3000 (Thermo Scientific). Luciferase activity was assessed using the pierce luciferase assay glow kit (Thermo Scientific), post‐transfection. Total luciferase activity of each well was normalized to protein level. Protein concentration was determined using a BCA assay (Thermo Scientific).

### 2.6 Data analysis and statistics

Data were analyzed using GraphPad software to determine significance between multiple treatments, we used a one‐way ordinary ANOVA with a Bonferroni post hoc test. A Student's *t* test was used to determine statistical significance between two groups. Unless otherwise stated, standard error of the mean is shown.

## 3 RESULTS

We previously showed that the canonical HDACi, trichostatin A (TSA), causes a significant increase in expression of the SUR2 (*Abcc9*) subunit of the K_ATP_ channel. Interestingly, we found that cholesterol concentration was reduced in TSA‐treated cells and addition of cholesterol to the culture medium inhibited the effect of TSA without affecting histone acetylation (Fatima et al., 2015). This result implied that there may be a cholesterol‐sensitive mechanism activated by HDACis that is required for increased expression of SUR2 in addition to the transcriptional effects of increased histone acetylation. Therefore, we tested the hypothesis that TSA increases SUR2 expression through a mechanism that requires cholesterol depletion and activation of the cholesterol sensitive SRE binding protein (SREBP) signaling pathway.

### 3.1 TSA treatment causes a decrease in cellular cholesterol and an increase in SUR2 mRNA expression

**Figure 1:**
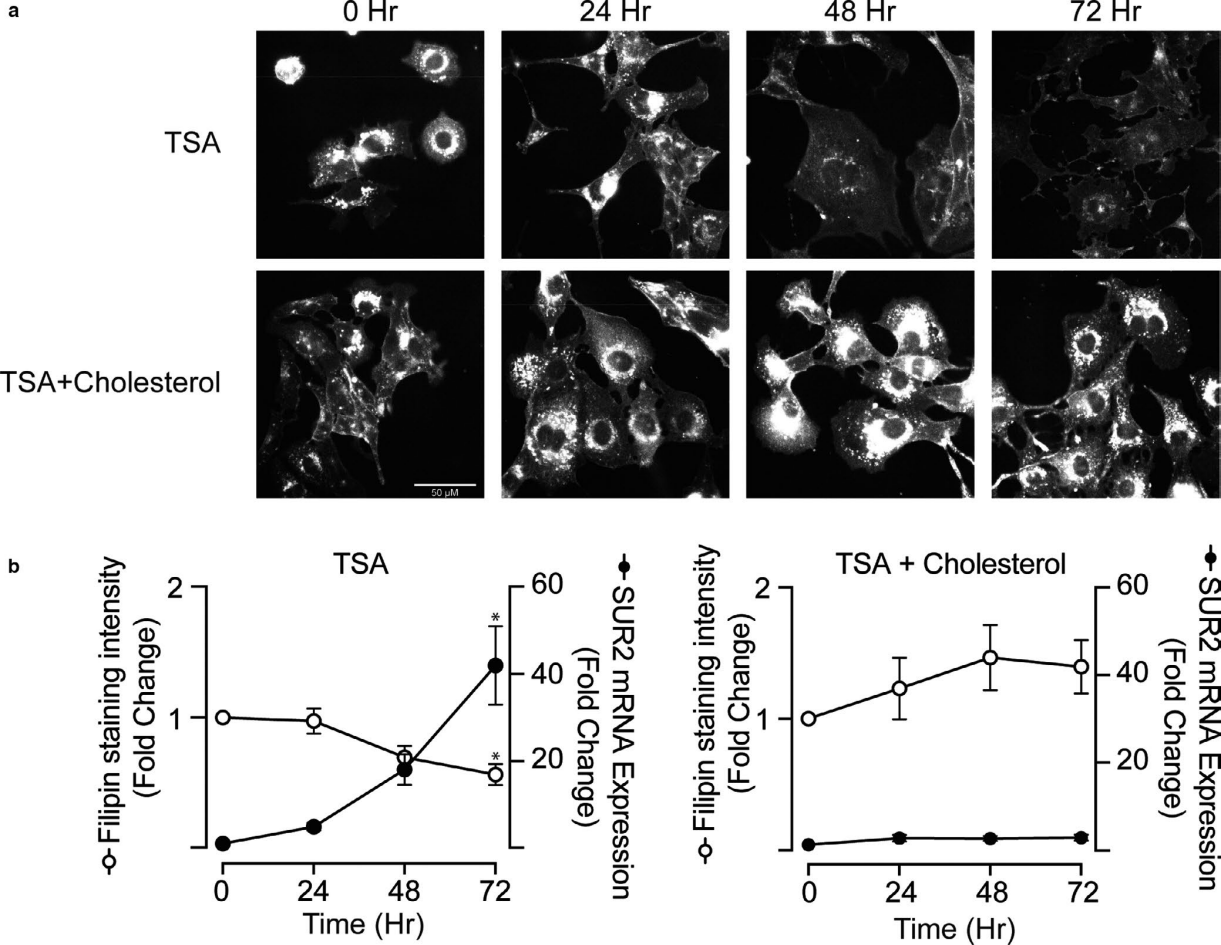
TSA‐dependent increase in SUR2 expression mirrors the time‐dependent reduction in cellular cholesterol. (a) Representative micrographs of HL‐1 cells following treatment with either TSA or TSA+Cholesterol for up to 72 hr. Cells were labeled with the fluorescent antibiotic filipin (0.05 mg/ml) to estimate cellular cholesterol concentration. (b) Summary data showing an inverse correlation of the cellular cholesterol (filipin staining intensity; n = 4 biological replicates, 14–62 per time point) and SUR2 subunit expression (n = 3; *p* < 0.05 ANOVA; Bonferroni Post hoc test *p < 0.05, 0 hr vs. TSA 72 hr) measured in parallel experiments in cells treated with TSA or TSA+cholesterol for the indicated duration. The addition of cholesterol inhibited both the decrease in cellular cholesterol and the increase in SUR2 expression.

To elucidate the role of cholesterol in regulating SUR2 expression, we first measured the time course of cellular cholesterol depletion and gene expression changes in TSA‐treated HL‐1 cardiomyocytes. To assess cellular cholesterol, we labeled cells with the fluorescent antibiotic, filipin (Leventhal et al., 2001). As expected if cellular cholesterol concentration falls, we observed a marked decrease in filipin staining following TSA treatment that is inhibited when exogenous cholesterol was also added to the media (Figure 1a). In parallel experiments, we measured SUR2 expression using qRT‐PCR (Figure 1b). We found that SUR2 mRNA expression is inversely correlated with the time‐dependent decrease in the cellular cholesterol concentration as estimated by filipin staining intensity. Addition of cholesterol to the media in the presence of TSA prevented both the decrease in filipin fluorescence and the increase in SUR2 expression. TSA alone or in the presence of cholesterol had little or no effect on SUR1 expression (*data not shown*). Taken together, the temporal correlation of SUR2 expression with the decrease in filipin staining suggests that cholesterol depletion in the presence of TSA is the principal catalyst for changes in SUR2 expression and supports the hypothesis that a previously unrecognized cholesterol‐sensitive mechanism may regulate K_ATP_ subunit expression.

### 3.2 TSA treatment causes cleavage of SREBP in HL‐1 cells

**Figure 2:**
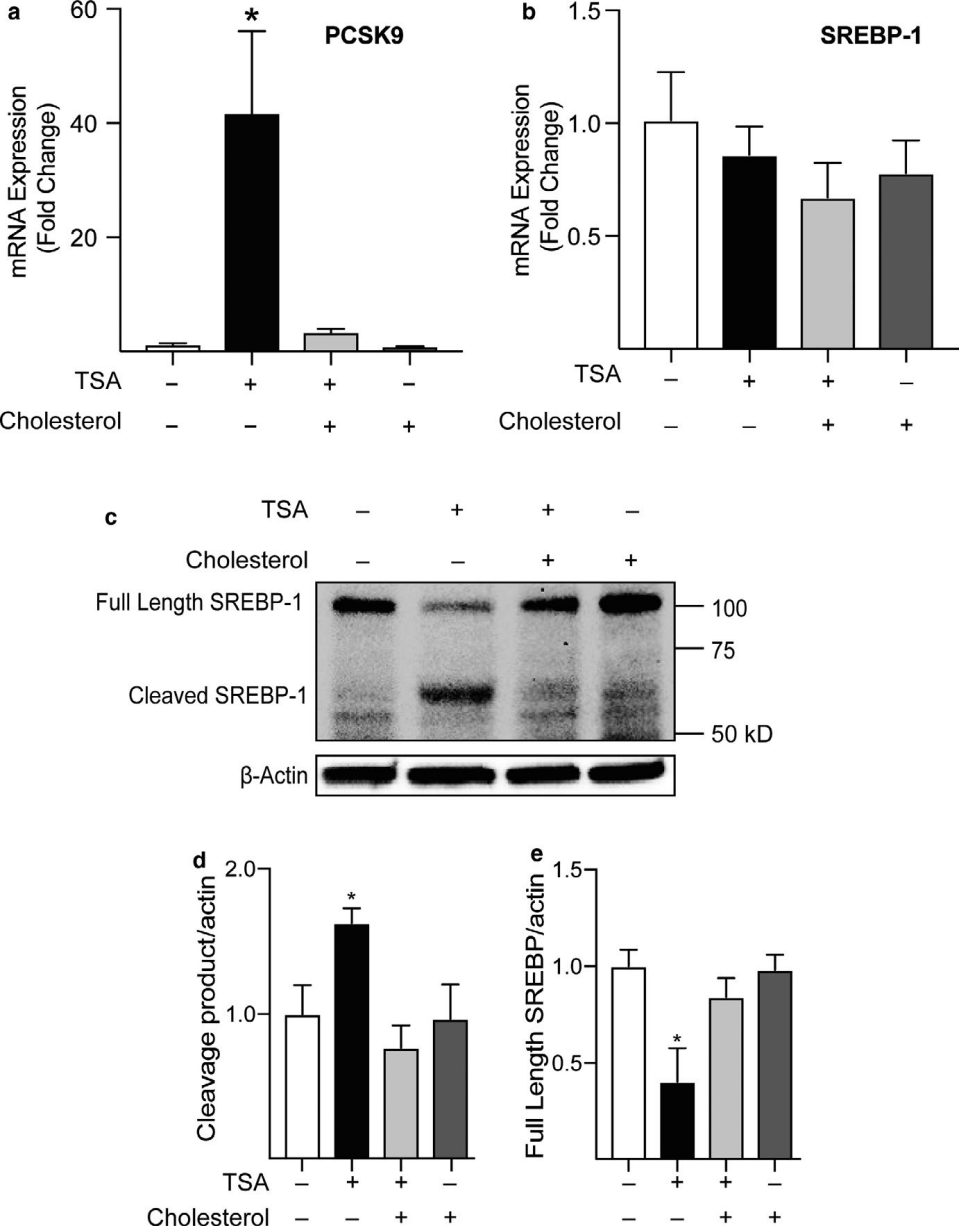
TSA treatment induces SREBP‐1 cleavage. (a) Relative mRNA expression of the SREBP target gene, Proprotein Convertase Subtilisin/Kexin type 9 (PCSK9), suggesting activation of SREBP signaling with TSA treatment (n = 4). (b) Relative SREBP‐1 mRNA expression did not increase in HL‐1 cells that were treated for 72 hr with TSA or cholesterol as indicated (n = 4). (c) Representative western blot (anti‐SREBP1; SC, cat no. sc‐365513) of whole‐cell lysates from HL‐1 cells treated for 72 hr with TSA or cholesterol as indicated. Summary quantitation of cleaved SREBP‐1 (d) and full‐length SREBP‐1 (e) protein from blots as in (b). There was an increase in cleaved and concomitant decrease in full‐length SREBP‐1 was only evident following treatment with TSA (n = 5; *p* < 0.05 ANOVA; Bonferroni Post hoc test *p < 0.05 TSA vs. control).

SREBP is a cholesterol‐sensitive transcription factor that is known to upregulate genes involved in cholesterol and fatty acid biosynthesis (Brown & Goldstein, 1999; Horton et al., 2002; Wang et al., 1994). For example, proprotein convertase subtilisin/kexin type‐9 (PCSK9) is an important regulator of cholesterol uptake and is a well‐characterized SREBP target gene (Jeong et al., 2008; Joseph & Robinson, 2015). In support of the hypothesis that TSA induces SREBP signaling, we observed a marked increase in PCSK9 mRNA expression in TSA‐treated cells, which was inhibited by cholesterol (Figure 2a). Because histone acetylation is associated with increases in gene expression through epigenetic mechanisms, we first determined whether TSA activated SREBP by increasing its expression. SREBP‐1 mRNA was not increased (Figure 2b), therefore, we next examined whether SREBP signaling was activated in TSA‐treated HL‐1 cells. When cholesterol levels in the cell are sufficient, SREBP is localized to the endoplasmic reticulum. If cellular cholesterol falls, SREBP is trafficked to the Golgi, where it is cleaved to form the active SREBP transcription factor (Wang et al., 1994). Indeed, in western blots we observe a significant increase in the presence of the SREBP cleavage product (~68 kDa) indicating cleavage and a corresponding decrease in the amount of full‐length SREBP‐1 in TSA‐treated cell lysates compared to control (Figure 2c–e). Moreover, when cells are treated with TSA and cholesterol, SREBP‐1 cleavage is inhibited, consistent with the conclusion that the TSA‐dependent fall of cellular cholesterol leads to cleavage and activation of SREBP signaling.

### 3.3 TSA treatment causes trafficking of SREBP to the nucleus of HL‐1 cells

**Figure 3:**
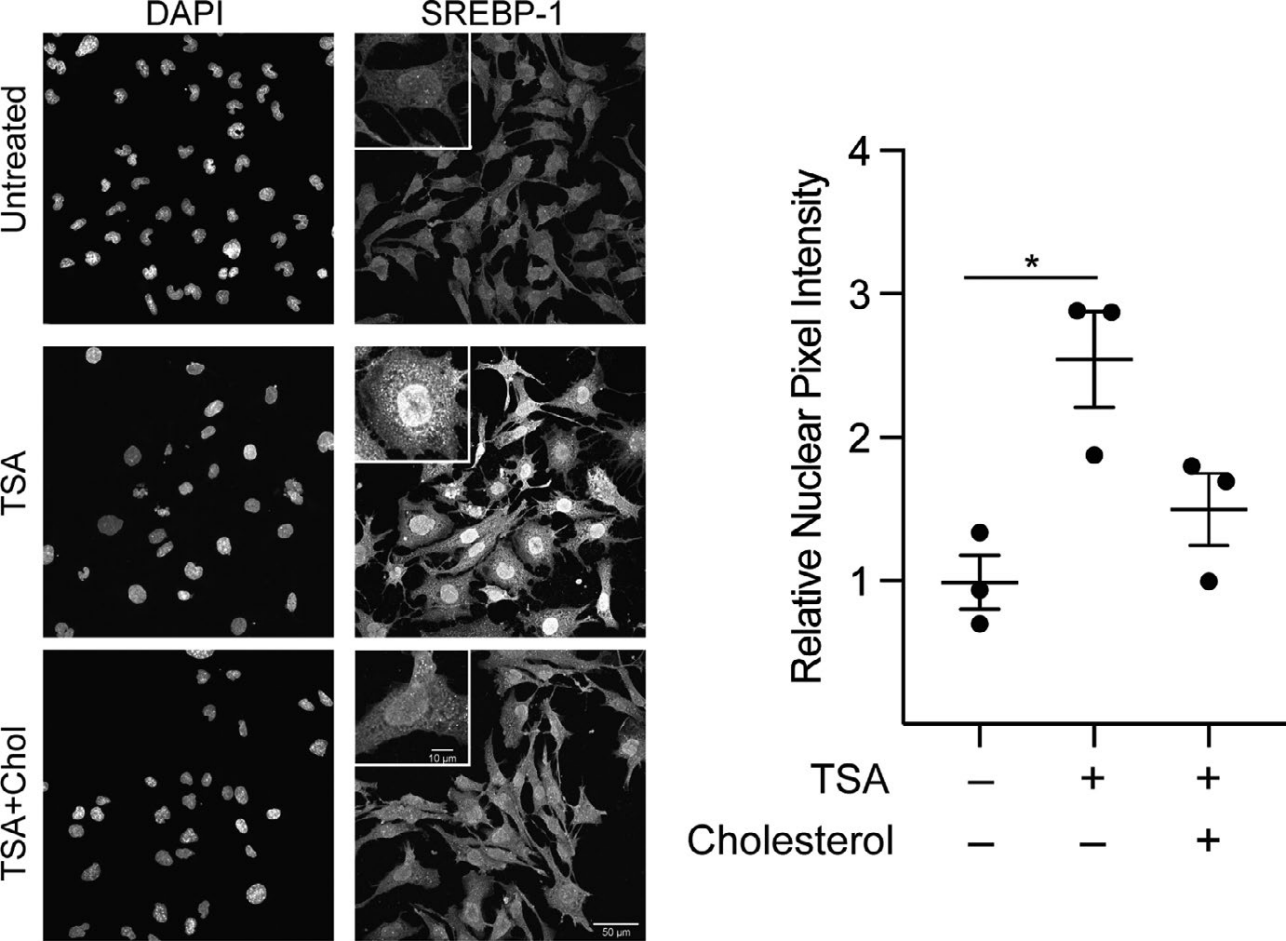
TSA treatment induces nuclear translocation of SREBP‐1. Images shown are representative Z‐projections of HL‐1 cardiomyocytes treated for 72 hr with TSA and cholesterol as indicated. Cells were labeled with both DAPI (*left*) and anti‐SREBP‐1 (*right*) (Abcam, cat no. ab28481). As highlighted in the inset, nuclear staining of SREBP‐1 was significantly greater in TSA‐treated cells compared to control and markedly reduced when cholesterol was added. Summary data for mean nuclear pixel intensity are shown at the right. Each symbol (●) reflects the mean of a single experiment in which 9–34 individual cells were analyzed for nuclear SREBP staining. The horizontal line corresponds to the mean ± SEM (n = 3; *p* < 0.05 ANOVA; Bonferroni Post hoc test *p < 0.05).

Cleavage of SREBP frees the truncated protein so that it can traffic to the nucleus and bind SREs in the genome (Wang et al., 1994; Yokoyama et al., 1993). Because TSA increases SREBP cleavage, it is expected that there will be more SREBP protein localized to the nucleus. To test this, we performed immunolocalization studies to determine whether TSA increased the amount of SREBP protein found in the nucleus (Figure 3). Indeed, HL‐1 cells treated with TSA showed a significant increase in SREBP‐1 localization to the nucleus when compared to either control cells or cells treated with both TSA and cholesterol. This result demonstrates that in addition to cleavage in the presence of TSA, SREBP protein translocates to the nucleus where it can stimulate gene transcription.

### 3.4 SREBP increases expression from the SUR2 promoter

**Figure 4:**
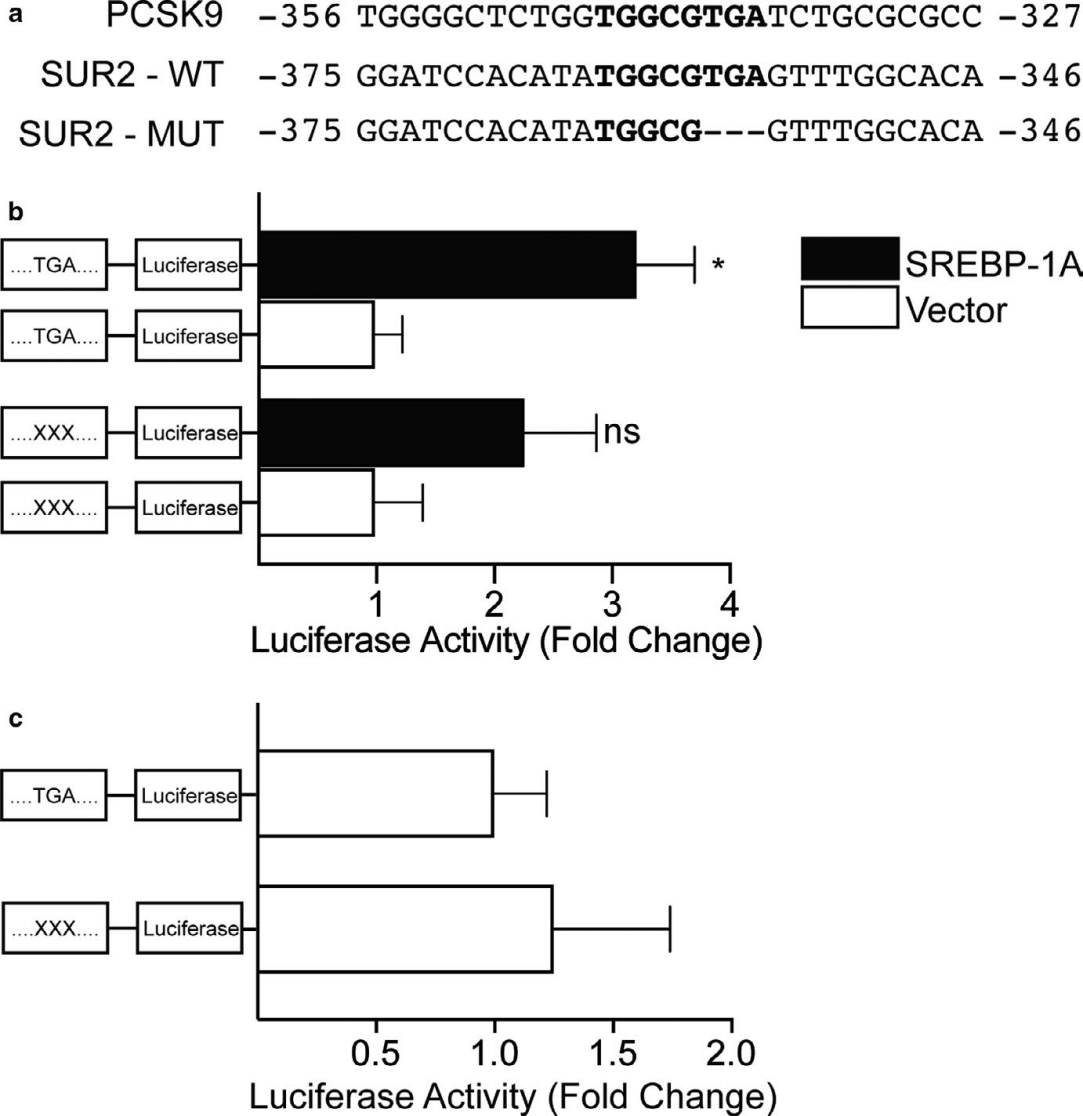
SREBP‐1a can activate the SUR2 promoter. (a) 470 bp of the noncoding sequence immediately upstream of the SUR2 transcription start site were cloned into a *Gaussia* luciferase reporter vector. Analysis of this sequence revealed a putative SREBP binding site in the antisense strand (position −356 to −365 relative to the transcription start site) similar to that found in the PCSK9 gene (Hyun et al., 2008). A mutant SUR2 sequence was constructed by deleting three nucleotides shown to be involved in SREBP binding (Magañ & Osborne, 1996). (b) Relative luciferase activity measured in COS‐m6 cells transfected with luciferase reporter constructs. Co‐transfection with SREBP‐1a induced a significant increase in relative luciferase activity compared to co‐transfection with vector control. Luciferase activity is not significantly increased when the putative SREBP binding site is mutated, while basal promoter activity (c) was not significantly changed by the mutation (n = 5–10, *p < 0.05 Student's *t* test).

We next sought to determine whether SREBP‐1 can increase transcription from the SUR2 promoter. To accomplish this, we cloned 470 bp of the noncoding region upstream of the SUR2 gene transcription start site into a plasmid encoding a *Gaussia* luciferase reporter gene. *In silico* analysis of this segment of the upstream regulatory sequence of the mouse SUR2 gene using Genomatix software (Cartharius et al., 2005) predicted the presence of an SREBP binding site, similar to the SRE in the PCSK9 gene (Hyun et al., 2008). This is also in agreement with similar data reporting the presence of an SREBP binding sequence in the promoter of both human and rat SUR2 genes (Philip‐Couderc et al., 2008) (Figure 4a). Previous studies have shown that deletion of the TCA nucleotides of the consensus SRE can interfere with SREBP binding and transactivation (Magañ & Osborne, 1996), therefore, we also constructed a mutant luciferase reporter construct where these nucleotides were deleted. Co‐transfection of pcDNA3.1‐2xFLAG‐SREBP‐1a (Toth et al., 2004) with the luciferase reporter containing the SUR2 promoter segment causes a three‐fold increase in luciferase activity (Figure 4b), while co‐transfection with the mutant luciferase reporter did not induce a significant increase in luciferase activity. Basal promoter activity was unaffected by the mutation (Figure 4c). These results indicate that the SUR2 promoter responds to activation of the SREBP signaling pathway, suggesting that SUR2 is a novel SREBP target gene.

### 3.5 The SREBP inhibitor PF429242 suppresses the TSA‐induced increase in SUR2 expression

**Figure 5:**
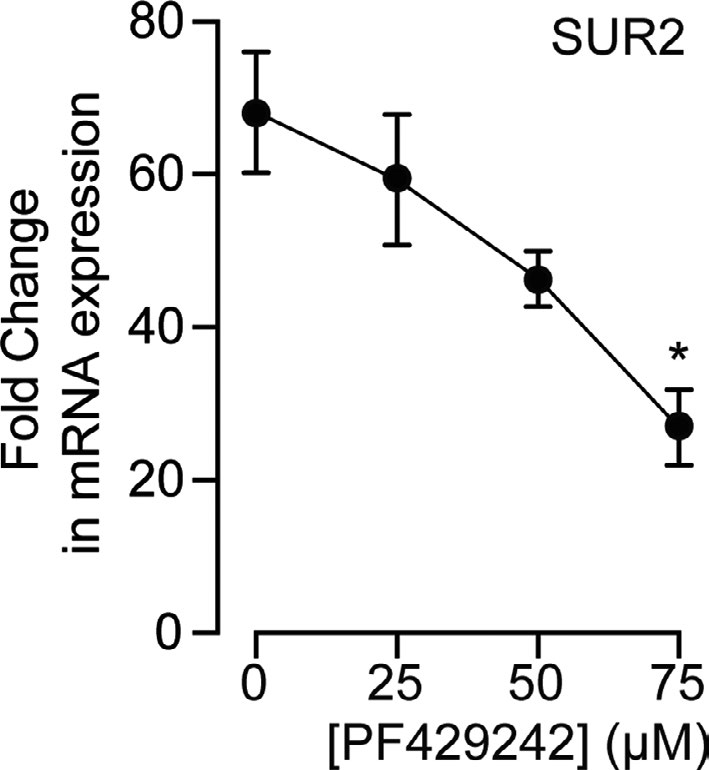
The SREBP inhibitor PF429242 the effect of TSA on SUR2 expression. Relative mRNA expression in HL‐1 cells treated with both TSA and different concentrations of the reversible competitive inhibitor of SREBP maturation, PF429242 (n = 4–6; *p* < 0.05 ANOVA; Bonferroni Post hoc test *p < 0.05 TSA vs. TSA+PF429242 75 μM).

Finally, to determine whether activation of SREBP signaling drives the increased SUR2 expression with TSA treatment, we used PF429242, a reversible competitive inhibitor that has previously been shown to reduce SREBP maturation (Hawkins et al., 2008; Sekar et al., 2017). PF429242 significantly inhibited the effect of TSA on SUR2 mRNA expression in a concentration‐dependent manner (Figure 5), consistent with the conclusion that the increase in SUR2 expression following TSA treatment results from cholesterol depletion and SREBP activation.

## 4 DISCUSSION

The present studies show that HDACi, like TSA, reduce cellular cholesterol activating a cholesterol‐dependent mechanism that regulates expression of K_ATP_ channel subunits. Indeed, the data show that the TSA‐induced increase in SUR2 gene expression correlates with cellular cholesterol levels and that SREBP is cleaved and translocated to the nucleus. Moreover, we show that suppression of SREBP signaling with PF429242 significantly reduces the TSA‐dependent increase in SUR2 expression. When coupled with data from luciferase reporter assays, these results demonstrate that cardiac K_ATP_ subunit expression can be directly regulated by cholesterol‐dependent activity of SREBP.

### 4.1 Cholesterol and K_ATP_ in the cardiovascular system

Elevated serum cholesterol is associated with poor cardiovascular health (Benjamin et al., 2019; Parish et al., 2012) and the development of hypertension (Halperin et al., 2006). Most consequences of high cholesterol are attributed to the development of arterial atherosclerotic plaques with consequent hypertension and increased risk for myocardial ischemia (Parish et al., 2012). Other findings associated with hypercholesterolemia are not as easily attributable directly to atherosclerosis and suggest that excess cholesterol may inhibit the function or expression of K_ATP_ channels. For example, hypercholesterolemia has been shown to impair ischemic preconditioning and the response to ischemia reperfusion (Csonka et al., 2014; Ferdinandy et al., 1997; Golino et al., 1987; Görbe et al., 2011; Kocić et al., 1999; Kyriakides et al., 2002; Maczewski & Maczewska, 2006; Osipov et al., 2009; Szilvassy et al., 1995; Ungi et al., 2005), both of which require the sarcolemmal K_ATP_ (Cole et al., 1991; Du et al., 2006; Gross & Auchampach, 1992; Suzuki et al., 2002; Wojtovich et al., 2013). In addition, the cholesterol‐reducing drug, simvastatin, reduces the no‐reflow area following myocardial infarction through a K_ATP_‐dependent mechanism with an increase in SUR2 expression (Yang et al., 2007). Here, we present a possible explanation for this finding, where reduction in cellular cholesterol activates SREBP signaling and increases K_ATP_ subunit expression. The present study is focused on cardiomyocytes, however, SUR2 is also a component of K_ATP_ channels in vascular smooth muscle (Chutkow et al., 2002; Morrissey et al., 2005; Yoshida et al., 2004). Given the importance of K_ATP_ in regulating blood pressure (Chutkow et al., 2002; Huang et al., 2018; Li et al., 2013; Miki et al., 2002), it is intriguing to speculate that cholesterol‐dependent reduction in SUR2B expression in vascular smooth muscle contributes to the hypertensive phenotype associated with hypercholesterolemia.

### 4.2 Cholesterol and ion channel regulation

Cholesterol is critical component lipid of the cell membrane and there have been a number of studies examining the role of cholesterol in modulating ion channel function (Levitan et al., 2014). For example, cholesterol loading inhibits recombinant Kir2.1 channels expressed heterologously in CHO cells, while cholesterol depletion increases current (Romanenko et al., 2004). This appears to be due to direct interaction of cholesterol with residues in the cytoplasmic domain of the channel (Epshtein et al., 2009). Studies also show that targeting to cholesterol‐enriched membrane domains is critical for normal regulation of K_ATP_ channel function by protein kinase A (Sampson et al., 2004). In the present study, we propose that cholesterol regulates ion channel subunit transcription through SREBP signaling. This is analogous to studies showing that Kir3.1 expression is increased in chick cardiomyocytes incubated in lipoprotein‐depleted medium as a result of SREBP activation (Park et al., 2008). SREBP has principally been associated with regulating the genes required for fatty acid and cholesterol synthesis and transport, however, it is increasingly recognized that the importance of SREBP is not limited to lipid homeostasis (Jeon & Osborne, 2012; Shao & Espenshade, 2012). Indeed, the results presented here add to this growing body of literature linking cholesterol and SREBP signaling with nonmetabolic physiological outcomes.

### 4.3 Cholesterol and arrhythmia

Increased K_ATP_ activity accelerates repolarization, reduces the refractory period, and predisposes to re‐entrant arrhythmias (Billman, 2008). It was recently demonstrated that trigger induced ventricular tachycardia or fibrillation during ischemia was significantly reduced in hypercholesterolemic mice (Ldlr^−/−^ or ApoA1^−/−^) (Baartscheer et al., 2015). This result of that study is consistent with the present study indicating that cholesterol suppresses K_ATP_ expression by inhibiting SREBP. Countering this observation, treatment with cholesterol‐lowering statins is associated with reduced arrhythmia risk (Buber et al., 2012). K_ATP_ channel activation can also stabilize the membrane potential and prevent triggered arrhythmias. It is possible that statin‐induced cholesterol reduction increases K_ATP_ expression to prevent arrhythmias, however, it is difficult to discern since statins are known to have pleiotropic effects variously attributed to modulation of nitric oxide synthase, Rho/ROCK kinase, Rac, and PPARs (Oesterle et al., 2017). Importantly, statins may affect other ion channels as well. For example, simvastatin has been shown to partially reverse the reduction in Kv4.3 expression in hypertrophic cardiomyocytes (Su et al., 2012). Nevertheless, the observation that K_ATP_ expression can be regulated by cholesterol and SREBP signaling suggests another way in which hypercholesterolemia and statin therapy may affect the heart.

### 4.4 HDACi‐dependent depletion of cholesterol

TSA is a canonical HDACi that is known to increase histone acetylation generally and in HL‐1 cardiomyocytes specifically (Fatima et al., 2015). In the present study, we show that TSA causes a time‐dependent decrease in intracellular cholesterol. TSA has been previously shown to reduce cholesterol accumulation in mutant Neimann Pick Type C1 (NPC) fibroblasts and SH‐SY5Y cells (Nunes et al., 2013; Pipalia et al., 2011). The mechanism underlying cholesterol depletion is not clear. In SH‐SY5Y cells, it has been proposed that TSA modulates expression of cholesterol trafficking genes (Nunes et al., 2013). In addition, treatment of HepG2 cells with TSA causes reduced expression of 11 of the 15 genes involved in cholesterol biosynthesis suggesting an activation of SREBP signaling (Chittur et al., 2008). It remains unknown why TSA causes a reduction in cholesterol in HL‐1 cardiomyocytes. In principle, TSA may increase cholesterol export, reduce cholesterol synthesis, or reduce cholesterol uptake. Interestingly, we do observe a significant (41‐fold) upregulation of PCSK9. PCSK9 is a protease that serves as a chaperone for the LDLr causing it to be recycled from the plasma membrane and targeted for degradation (Joseph & Robinson, 2015). The use of PCSK9 inhibitors represents a new approach to lower cholesterol in patients with hypercholesterolemia, achieving significant reductions in circulating cholesterol by stabilizing LDLr in the hepatocyte membrane and increasing cholesterol uptake (Sabatine, 2019). It remains unknown how TSA causes cholesterol depletion in HL‐1 cells and additional studies will be needed to examine this interesting observation. Nevertheless, in the context of the present study, TSA is a potent molecular tool to deplete cellular cholesterol and reveals a novel cholesterol‐dependent mechanism of K_ATP_ subunit expression.

## 5 CONCLUSION

In summary, the results demonstrate that TSA causes a reduction in cellular cholesterol that activates SREBP signaling and increases SUR2 expression. It is known that aggressive cholesterol‐lowering therapies have significant benefit for patients with cardiovascular disease. We postulate that cholesterol lowering therapies would increase K_ATP_ as one of the many beneficial effects on the cardiovascular system. Moreover, because K_ATP_ channels protect the heart during metabolic stress, patients with hypercholesterolemia may not only be both more likely to experience an ischemic event due to atherosclerosis, but less able to cope with the ischemic event due to reduced K_ATP_ expression in the heart.

## DISCLOSURE

The opinions and assertions expressed herein are those of the author(s) and do not necessarily reflect the official policy or position of the Uniformed Services University or the Department of Defense. Neither I nor my family members have a financial interest in any commercial product, service, or organization providing financial support for this research.

## AUTHOR CONTRIBUTIONS

RG designed and performed experiments, analyzed the data, drafted and edited the manuscript. NF performed experiments and edited the manuscript. JFS maintained HL‐1 cells. JTS assisted with the design and analysis of imaging experiments. MCH assisted in data analysis and edited manuscript. TPF designed experiments, analyzed data, and edited the manuscript. All authors have read and approved the final manuscript.
